# Lignan-containing maple products inhibit *Listeria monocytogenes* biofilms on fresh produce

**DOI:** 10.3389/fmicb.2023.1258394

**Published:** 2023-10-19

**Authors:** Ahmed M. Elbakush, Alex M. Fulano, Mark Gomelsky

**Affiliations:** ^1^Department of Molecular Biology, University of Wyoming, Laramie, WY, United States; ^2^Faculty of Veterinary Medicine, University of Tripoli, Tripoli, Libya

**Keywords:** *Listeria* (*L*.) *monocytogenes*, listeriosis, biofilm, exopolysaccharide, fresh produce and foodborne illness, maple (*Acer*), *Carya*, lignan

## Abstract

Major listeriosis outbreaks have been associated with fresh produce contaminated with *Listeria monocytogenes*. Strains that synthesize the Pss exopolysaccharide (EPS) have an estimated 10^2^ to 10^4^-fold advantage over nonsynthesizing strains in causing listeriosis. They more readily attach to the surfaces of fruit and vegetables forming EPS-biofilms that better withstand stresses associated with produce storage and consumption. Here, we show that the threat to fresh produce safety posed by the listerial EPS-biofilms may be countered by broadly available maple products. We serendipitously discovered that aqueous extracts of wood from several *Acer* (maple) and *Carya* (pecan, hickory) species inhibit the formation of listerial EPS-biofilms without affecting bacterial viability. One active ingredient in maple wood was identified as nortrachelogenin-8’-O-β-D-glucopyranoside (NTG). At 120 μM, this lignan decreased colonization of the EPS-synthesizing *L. monocytogenes* on cantaloupe pieces by approximately 150-fold, and on cut celery and lettuce by 10 to 11-fold. Another lignan, lariciresinol, which is abundant in a common food sweetener, maple syrup, had antibiofilm activity comparable to that of NTG. Diluted in the range of 1:200 to 1:800 maple syrup from two random manufacturers prevented formation of listeiral EPS-biofilms. Importantly, not only did maple products drastically decrease colonization of fresh produce by the EPS-synthesizing strains, they also decreased, by 6 to 30-fold, colonization by the *L. monocytogenes* strains that do not synthesize measurable EPS, including strains from the infamous 2011 cantaloupe listeriosis outbreak. Inhibition of surface colonization by various listerial strains, broad availability of maple sap and syrup as well as maple lumber processing waste position maple products as potential antibiofilm agents for protecting fresh produce from *L. monocytogenes*.

## Introduction

Listeriosis outbreaks caused by fresh produce as a source of *Listeria monocytogenes* contamination have become common in the past decades (Zhu et al., 2017). Major listeriosis outbreaks worldwide have been associated with contaminated whole cantaloupes (also known as rock melons), cut celery, packaged salads, bean sprouts, caramelized apples, mushrooms, and frozen vegetables (CDC, 2022). While healthy individuals who consume food contaminated with *L. monocytogenes* experience relatively mild-to-moderate gastrointestinal problems, susceptible individuals often succumb to severe systemic illness, with extremely high, 15 to 20%, mortality rates in the Western countries (CDC, 2022). In the USA, a “zero tolerance” policy is implemented regarding the presence of *L. monocytogenes* in ready-to-eat food products (Archer, 2018), yet complete prevention of contamination is challenging due to the ubiquity of this bacterium in the environment. Contamination usually takes place postharvest due to the contaminated environment and equipment at the facilities where fresh produce is processed and stored (Ferreira et al., 2014; Garner and Kathariou, 2016; Rodríguez-López et al., 2018; Marik et al., 2020). Preventing *L. monocytogenes* contamination at such facilities would seem most effective, yet even harsh cleaning and disinfection methods often fail to remove listerial biofilms on hard-to-access pieces of equipment, such as conveyor belts (Fagerlund et al., 2020). A complementary approach would be to treat fresh produce with antibiofilm agents. However, at present, we do not fully understand the factors contributing to listerial colonization of fruit and vegetables and the factors affecting bacterial survival during fresh produce washing, disinfection, storage and transportation.

We have recently uncovered the Pss exopolysaccharide (EPS) as an underappreciated factor strongly promoting listerial colonization of plant surfaces (Fulano et al., 2023). The Pss EPS is synthesized by *L. monocytogenes* at high intracellular concentrations of the second messenger, c-di-GMP (Chen et al., 2014). The association of increased c-di-GMP levels with the EPS synthesis and the biofilm lifestyle is common to many species of bacteria (Römling et al., 2013). In *L. monocytogenes*, the Pss EPS promotes the colonization of fruit and vegetables but does not significantly affect colonization of the manmade materials, such as steel, glass or plastics. The extent of EPS-mediated colonization depends on the nature and roughness of plant surfaces. For example, the Pss increases listerial colonization of the smooth surfaces of cantaloupe flesh by 2 to 3-fold, whereas colonization of the netted surfaces of cantaloupe rind is increased by 12-fold (Fulano et al., 2023). From the standpoint of produce safety, not only primary attachment but also survival of bacteria within the EPS-containing biofilms is important, and survival is known to greatly improve due to enhanced tolerance of various stresses (Flemming et al., 2016). On the fresh produce surfaces, *L. monocytogenes* may experience desiccation during storage and transportation (Esbelin et al., 2018), and subsequently acid stress during passage through the stomach acid following consumption of the contaminated produce (Tennant et al., 2008). Survival of each of these stresses is increased by 6 to 100-fold in the Pss EPS-biofilms. We estimated that better surface colonization and higher survival of desiccation and acid stress, combined, give the EPS-synthesizing *L. monocytogenes* strains an enormous, 10^2^ to 10^4^-fold, advantage over the nonsynthesizing strains in reaching the small intestine of consumers where *L. monocytogenes* establishes infection (Fulano et al., 2023).

The Pss EPS is the only known cell-attached EPS synthesized by listeria. It is made of the repeating trisaccharide unit, {4)-β-ManpNAc-(1–4)-[α-Galp-(1–6)]-β-ManpNAc-(1-} (Köseoğlu et al., 2015). The biosynthetic pathway of this EPS is encoded in the *pss* operon, which is highly conserved among listerial strains. It belongs to the *L. monocytogenes* core genome derived from 1,696 genome sequences from the isolates from various sources and geographic locations (Moura et al., 2016). The *pss* operon is also ubiquitously present in non-*monocytogenes* species of *Listeria*, suggesting the importance of the Pss EPS for environmental survival (Chen et al., 2014). At present, we know little about factors promoting the Pss EPS synthesis, aside from the understanding that it requires high levels of the intracellular second messenger, c-di-GMP. Cyclic di-GMP acts via the PssE effector, which likely activates the PssC glycosyltransferase, the key synthetic enzyme in making the Pss polymer (Chen et al., 2014).

The Pss EPS hydrolase, designated PssZ, is involved in the pruning of the growing Pss chains during synthesis and in the hydrolysis of the pre-formed Pss EPS, a process necessary for bacterial escape from the EPS-biofilm matrix. When added exogenously, the PssZ hydrolase disperses EPS-biofilms. Therefore, PssZ can be used as an antibiofilm agent (Köseoğlu et al., 2015). However, the application of PssZ or other EPS hydrolytic enzymes at large scale may be prohibitively expensive. Therefore, finding abundant yet inexpensive natural products that inhibit the formation of the EPS-biofilms in *L. monocytogenes* and/or promote dispersion of the EPS-biofilms is of significant interest (Oloketuyi and Khan, 2017).

In this study, we report a serendipitous discovery of the listerial antibiofilm activity of certain wood products, particularly maple wood. The lignans present in maple wood extracts and maple syrup were found to possess potent antibiofilm activity that drastically decreases colonization of fresh produce by the *L. monocytogenes* strains that synthesize EPS and moderately decreases colonization by the strains that do not synthesize measurable EPS. This finding is intriguing because waste products from maple lumber processing such as wood shavings, chips, sawdust and bark are widely available. Further, maple sap and syrup are food products that are annually produced in millions of gallons (Statistics Canada, 2023; USDA, 2023). We suggest that abundance and lack of toxicity opens the possibility of using maple products for protecting fresh produce from listerial contamination.

## Results

### Maple wood contains an inhibitor(s) of listerial EPS-containing biofilms

**Figure 1:**
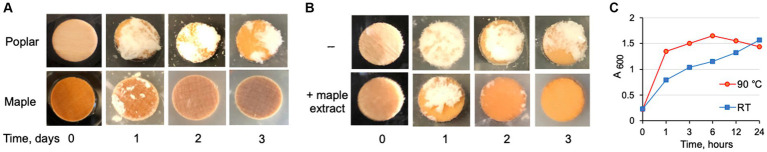
Inhibition of listerial EPS-biofilms on wood coupons by the maple compounds. **(A)** Observation of formation and dispersion of *L. monocytogenes* EPS-biofilms on wood coupons from poplar or maple incubated with the EPS-synthesizing strain, *ΔpdeB/C/D*, at 30°C. The EPS-biofilms on maple, but not poplar, coupons disappear after 2 days. Cell aggregates (clumps) in the medium (visible as white chunks on the black background) are dispersed over time in the presence of maple, but not poplar, coupons resulting in homogenous cell suspension (gray). **(B)** Addition of aqueous maple wood extract inhibits EPS-biofilms on poplar coupons and promotes clump dispersion. 1 mL extract (made from soaking 1 g maple coupons in 10 mL water) was added to 10 mL culture inoculated with the EPS-synthesizing strain. **(C)** Biofilm dispersion activity of aqueous maple wood extracts prepared by soaking maple chips at 90°C or at room temperature (RT) for the indicated duration of time. The antibiofilm activity is released faster at 90°C than at RT but over time reaches similar levels. Data from one representative experiment are shown.

In our recent study of the *L. monocytogenes* EPS-biofilms on the surfaces of fruit and vegetables, we grew strains with varying levels of EPS production in liquid medium in the presence of wood coupons (disks) as a proxy for the fresh produce pieces (Fulano et al., 2023). In these experiments, most indicative were biofilms formed by the EPS-producing strain, *ΔpdeB/C/D*, in which the Pss EPS synthesis is constitutively turned on due to high levels of the second messenger c-di-GMP (Chen et al., 2014). While we initially considered wood to be a neutral plant surface, we were surprised to find that biofilms formed by the EPS-synthesizing strain behaved differently in the course of incubation, dependent on the wood source (Fulano et al., 2023). While EPS-biofilm abundance on most wood coupons varied somewhat over time due to the mechanical shearing and changes in the composition of the growth medium (Figure 1A, poplar), biofilms formed on coupons from maple (genus *Acer*) were invariably dislodged after 48 h of incubation (Figure 1A, maple). Furthermore, the free-floating EPS-based cell aggregates (clumps) were dispersed, resulting in homogenous cell suspension (Figure 1A). These observations suggested that maple wood contains a compound(s) that inhibits listerial EPS-biofilm formation and/or promotes dispersion, which is exciting because maple wood and its products are abundant and thus may serve as an affordable source of listerial antibiofilm compounds.

We surmised that the antibiofilm compound(s) gradually leaks from the wood into the medium prior to reaching the active antibiofilm concentration. To test this hypothesis, we boiled maple wood coupons in water, and added the aqueous extract to the growth medium containing poplar coupons. Maple wood extract induced dispersion of the EPS-biofilms initially formed on the poplar coupons by the EPS-synthesizing strain and dispersed EPS-dependent clumps (Figure 1B), thus supporting our hypothesis.

To investigate whether the EPS biofilm inhibitor is present in the wood or whether it is derived from hydrolysis of the insoluble lignocellulosic polymers, we compared the maximum activities of the aqueous extracts derived from maple wood coupons that were soaked in water at 90°C or at room temperature. We expected high temperature treatment to yield higher levels of the inhibitor if it was derived from hydrolysis. However, soaking wood coupons at different temperatures generated extracts with very similar final antibiofilm activity (Figure 1C), albeit the maximum concentration was reached faster when wood was soaked at 90°C. This result indicates that the antibiofilm compound(s) is present in the maple wood in the active form and likely slowly leaches out from the wood.

### Maple wood extracts inhibit listerial EPS-biofilms on fresh produce

**Figure 2:**
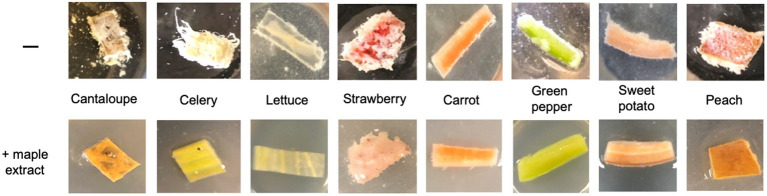
Formation of listerial EPS-biofilms on various fruit and vegetables is inhibited by the aqueous maple wood extract. Sterilized pieces of fresh produce were incubated at 30°C for 48 h in the minimal medium with the EPS-synthesizing strain, in the absence (“−”) or presence of aqueous maple wood extract (“+ maple extract”).

The presence of an antibiofilm compound in such an abundant source as maple wood encouraged us to evaluate the ability of maple wood extracts to protect fresh produce from *L. monocytogenes* biofilms. To this end, we incubated pieces of fresh produce with the EPS-synthesizing *L. monocytogenes* strain, *ΔpdeB/C/D*, in the presence or absence of aqueous maple wood extracts. We assayed rind-containing pieces of cantaloupe, as well as pieces of celery and lettuce, which we tested for colonization by the EPS-synthesizing listeria earlier (Fulano et al., 2023). We also assayed new kinds of fruit and vegetables, including strawberry, peach, carrot, green pepper, and sweet potato. The EPS-synthesizing strain readily attached to the surfaces of all tested fresh produce forming visible biofilms, which is consistent with the notion that the Pss EPS promotes attachment to plant surfaces via interactions with the lignocellulosic polymers, i.e., rather nonspecifically (Fulano et al., 2023). The addition of maple wood extracts drastically decreased biofilm formation on all tested produce (Figure 2), suggesting that the antibiofilm activity is independent of the produce type.

### Wood extracts from many *Acer* and *Carya* species possess the *Listeria monocytogenes* antibiofilm activity

To investigate whether the antibiofilm activity is unique to a particular species of maple, we prepared aqueous extracts from the dried stems collected from tree nurseries and regionally growing maple trees. The extracts were made from stems of red (*A. rubrum*), silver (*A. saccharinum*), boxelder (*A. negundo*) and Norway (*A. pseudoplatanus*) maple (1 g dried stems soaked in 10 mL water) and added (1 mL extract per 10 mL medium) to the cultures of the EPS-synthesizing *L. monocytogenes* strain. The extracts from all tested maple samples inhibited clumping (not shown), while, predictably, the degree of inhibition varied somewhat dependent on the sample. These experiments allowed us to conclude that the *L. monocytogenes* antibiofilm activity is present in various maple species.

**Figure 3:**
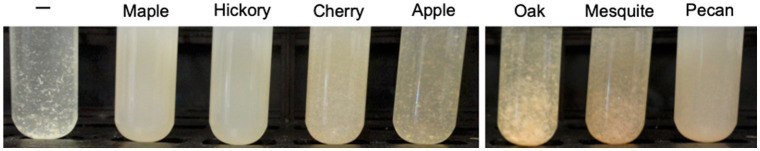
Inhibition of *L. monocytogenes* EPS-clump by the aqueous wood extracts from selected tree species. High listerial antibiofilm activity, manifested as the lack of clumps, is evident in the presence of aqueous extracts from maple wood as well as wood of *Carya* species, hickory and pecan. Extracts (1 g wood chips per 10 mL water) were made from commercially purchased wood chips soaked in water for 24 h and added (1 mL per 10 mL culture) to the 125-mL flasks inoculated with *L. monocytogenes ΔpdeB/C/D*. After 48-h growth, cultures were transferred to glass tubes and photographed.

Next, we assessed whether the antibiofilm activity was uniquely present in maple wood. To address this question, we incubated the EPS-synthesizing *L. monocytogenes* strain in the presence of aqueous extracts made from wood chips of several non-maple trees. Significant antibiofilm activity, measured as inhibition of clumping, was evident in extracts derived from hickory and pecan. Little or no activity was observed in extracts derived from oak, mesquite, apple and cherry trees (Figure 3). Both hickory and pecan belong to the *Carya* genus. While we did not test whether the same or different compounds are responsible for the observed antibiofilm activity in *Acer* and *Carya* genera, it appears that such compounds are not unique to maple.

### Lignan nortrachelogenin-8’-O-β-D-glucopyranoside (NTG) is one of the antibiofilm compounds in maple wood

**Figure 4:**
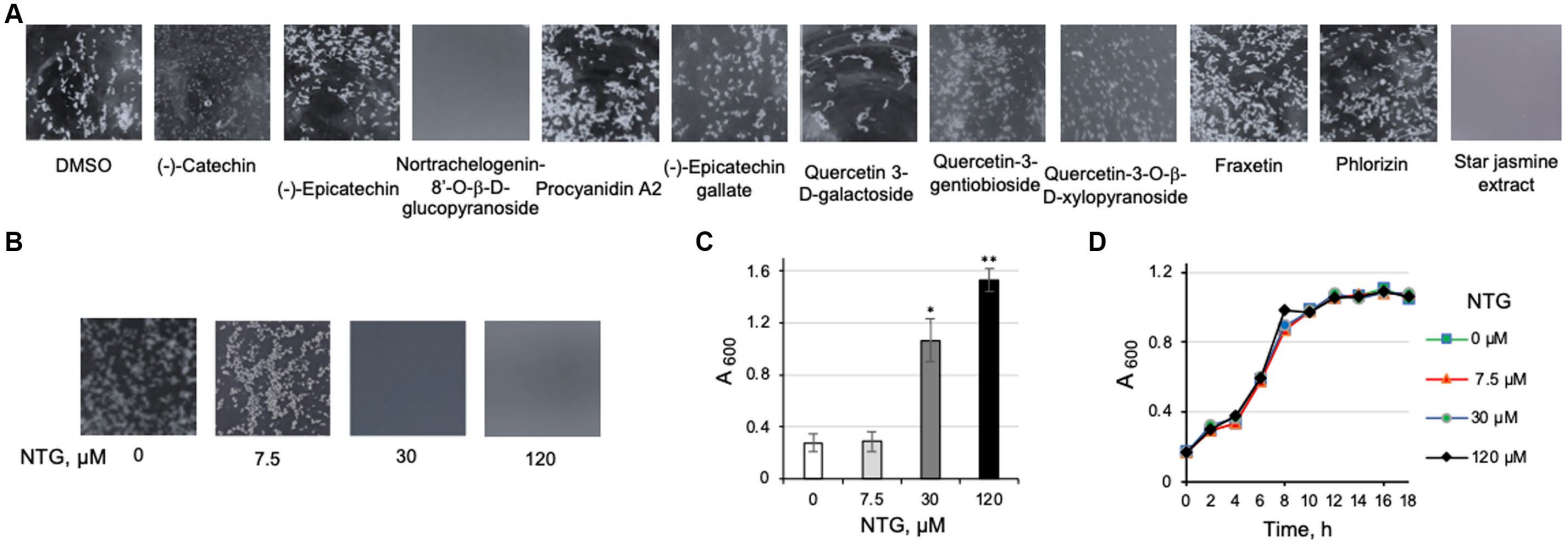
Identification and characterization of nortrachelogenin-8’-O-β-D-glucopyranoside (NTG) as an antibiofilm compound from maple. **(A)** The effect of the polyphenol components reported to be present in maple wood aqueous and methanol extracts on clump dispersal of the *L. monocytogenes ΔpdeB/C/D* strain. NTG possesses the highest antibiofilm activity. Cultures were grown for 48 h in the presence of the tested compounds (120 μM, final concentration). Aliquots were poured into a small Petri dish and photographed on the black background. DMSO, a solvent for all tested compounds, was used as negative control. Homogenous gray background due to complete clump dispersal signifies high antibiofilm activity. Aqueous extract from the stems of star jasmine (*T. jasminoides*), the source of commercial NTG, also possess potent listerial antibiofilm activity. Because a commercial source of quercetin-3-O-(2”-O-galloyl)-α-L-rhamnopyranoside reported in the maple wood methanol extract (Wan et al., 2012) could not be found, three related quercetin derivatives were tested. **(B)** Dose-dependent inhibition of *L. monocytogenes ΔpdeB/C/D* clumping by NTG using the same setup as in panel **(A)**. **(C)** Quantification of the effect of NTG via a clump precipitation assay. *L. monocytogenes ΔpdeB/C/D* was grown for 48 h in the presence of varying levels of NTG. Higher A_600_ corresponds to higher antibiofilm activity. **(D)** Growth curves of *L. monocytogenes* EGD-e in the presence of NTG showing that NTG does not impair listerial viability at the highest tested concentrations.

To identify potential antibiofilm compounds in maple, we looked for literature reports describing water-and methanol-soluble compounds because methanol as solvent resembles water most closely, compared to less hydrophilic solvents previously used for extraction. None of the water-and methanol-soluble nonphenolic and simple phenolic compounds tested (Wong et al., 2005; Wan et al., 2012) possess antibiofilm activity, including sugars (sucrose, raffinose, stachyose, glucose, fructose, xylose), organic acid anions (malate, citrate, succinate), and simple phenolics (dihydroxybenzoate, hydroxyphenylacetate, methyl gallate, *p*-coumarate, vanillate). Among polyphenolic compounds reported to be most abundant in the maple wood methanol extracts (Wan et al., 2012), one, NTG, possessed antibiofilm activity at 120 μM (Figure 4A). NTG belongs to lignans, a rare class of polyphenolic chemicals in the maple methanol extract. NTG inhibited clumping of the EPS-synthesizing strain in liquid culture in a dose-dependent manner (Figures 4B,C). Similar to the maple extract, NTG did not impair the viability of listeria (Figure 4D), suggesting that it interacts with a specific target in *L. monocytogenes* involved in EPS formation and/or dispersion.

For commercial purposes, NTG is purified from the *Trachelospermum* plants (family *Apocynaceae*) (Tan et al., 2005; Zhao et al., 2017). We therefore expected that aqueous extracts prepared from *Trachelospermum jasminoides*, commonly known as star jasmine or Luoshiten in Chinese folk medicine, will inhibit *L. monocytogenes* EPS-biofilms. To test this prediction, we soaked the dried stems of a *T. jasminoides* plant (1 g per 10 mL water) purchased from an online nursery and added the aqueous star jasmine extract to the culture of the EPS-synthesizing strain. Consistent with our expectations, the star jasmine extract completely inhibited clumping at lower amounts (0.3 mL extract per 10 mL culture) then the amounts of maple wood extracts (Figure 4A).

**Figure 5:**
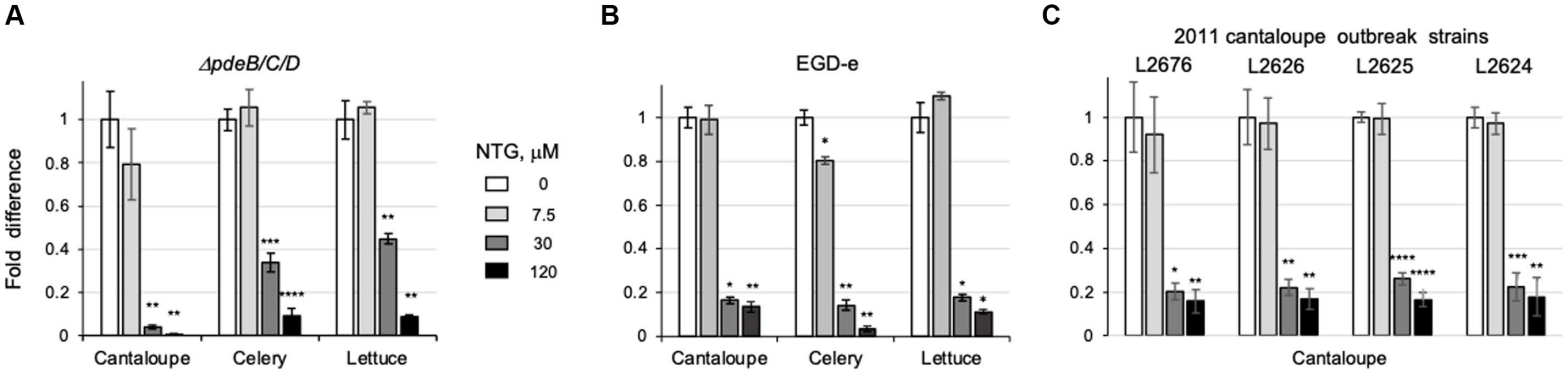
NTG protects fresh produce from *L. monocytogenes* EPS-biofilms. **(A)** Comparison of the CFUs in biofilms formed by the *L. monocytogenes ΔpdeB/C/D* strain on pieces of cantaloupe, celery and lettuce in the presence of varying levels of NTG. CFUs were evaluated after 48-h incubation in the HTM/G medium. Shown are fold-differences compared to the CFUs in the absence of NTG. The average CFUs in the absence of NTG were 5.2 × 10^10^ per cantaloupe piece, 5.1 × 10^9^ per piece of celery, and 6.9 × 10^9^ per piece of lettuce. Error bars represent standard deviations from three independent experiments with three pieces in each experiment. **(B)** Colonization of cantaloupe pieces by the EGD-e strain. Conditions are the same as in panel **(A)**. The average CFUs in the absence of NTG were 3.4 × 10^9^ per cantaloupe piece, 7.6 × 10^8^ per piece of celery, and 1.1 × 10^9^ per piece of lettuce. **(C)** Colonization of cantaloupe pieces by the 2011 whole cantaloupe outbreak strains. The average CFUs in the absence of NTG were 3.4 × 10^9^ for L2676, 3.6 × 10^9^ for L2626, 3.5 × 10^9^ for L2625, and 3.6 × 10^9^ for L2624.

Next, we tested if NTG inhibits EPS-biofilms on the surfaces of pieces of cantaloupe containing rind, celery or lettuce. As shown in Figure 5A, the addition of NTG decreased, in a dose-dependent manner, the number of colony forming units, CFUs, on all tested pieces of produce. NTG at 7.5 μM was largely ineffective, at 30 μM it inhibited produce colonization partially, and at 120 μM completely. The CFUs on cantaloupe pieces exposed to 120 μM NTG decreased by >158-fold (from 5.2 × 10^10^ to 3.3 × 10^8^), on cut celery – by >10-fold (from 5.1 × 10^9^ to 4.7 × 10^8^); and on lettuce – by >11-fold (from 6.9 × 10^9^ to 6.0 × 10^8^).

### NTG inhibits colonization of fresh produce surfaces by various *Listeria monocytogenes* strains

We wondered whether NTG-dependent inhibition of *L. monocytogenes* colonization of plant surfaces would affect strains that do not synthesize high levels of EPS. To address this question, we assayed the effect of NTG on cantaloupe colonization by the wild-type strain, EGD-e, from which the *ΔpdeB/C/D* mutant is derived. EGD-e does not synthesize measurable amounts of EPS under the experimental conditions used here (Chen et al., 2014; Fulano et al., 2023). As anticipated from our earlier report (Fulano et al., 2023), in the absence of NTG, the EGD-e strain colonized fresh produce pieces significantly less efficiently than the EPS-synthesizing *ΔpdeB/C/D* strain, i.e., its CFUs were ~ 15-fold lower on cantaloupe pieces (3.4 × 10^9^ versus 5.2 × 10^10^), by ~7-fold lower on celery (7.6 × 10^8^ versus 5.1 × 10^9^) and by ~6-fold lower on lettuce (1.1 × 10^9^ versus 6.9 × 10^9^). In the presence of 120 μM NTG, the numbers of EGD-e bacteria on the produce pieces were decreased by ~7-fold on cantaloupe, by ~30-fold on celery and by ~8-fold on lettuce, compared to the absence of NTG (Figure 5B). These results show that, while the effect of NTG inhibition on produce colonization is most pronounced in the EPS-synthesizing strain, NTG inhibits colonization even in the absence of EPS.

Next, we tested if NTG inhibits cantaloupe colonization in the strains associated with the 2011 whole cantaloupe listeriosis outbreak, one of the deadliest foodborne pathogen-associated outbreaks in modern US history (McCollum et al., 2013). Four strains isolated from that outbreak were tested. As shown in Figure 5C, all four strains colonized cantaloupe pieces with similar efficiency, i.e., (3.4–3.6) × 10^9^ CFUs per piece, that was comparable to that of EGD-e (3.4 × 10^9^). At 120 μM NTG, CFUs on cantaloupe pieces in the cantaloupe outbreak strains were decreased by 6 to 7-fold, similar to the decrease observed in the EGD-e strain. These results suggest that the target of NTG is common to *L. monocytogenes* strains. Therefore, NTG and aqueous extracts from maple wood may be used to lower contamination on fresh produce by various listerial strains.

### Diluted maple syrup inhibits *Listeria monocytogenes* biofilms

**Figure 6:**
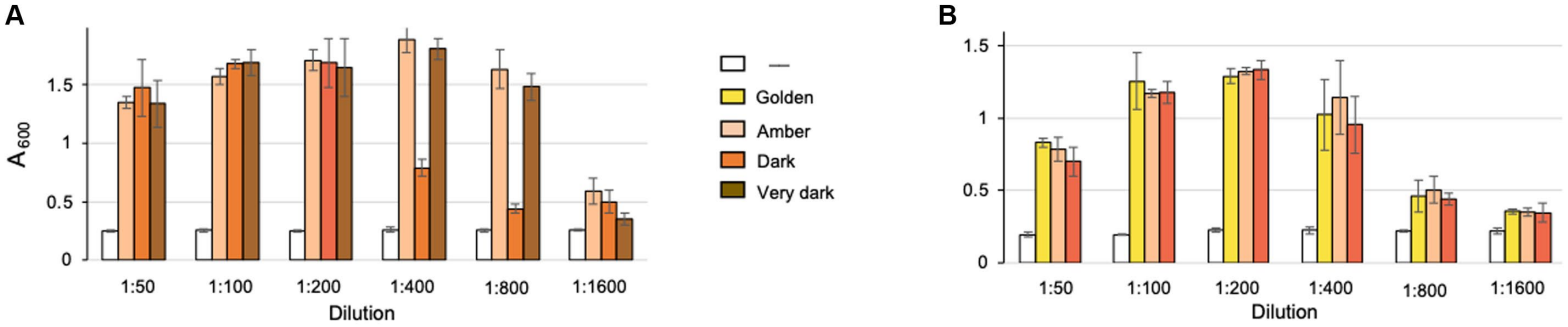
Effect of diluted maple syrup on *L. monocytogenes* biofilm inhibition. Shown are results of the clump precipitation assays. Maple syrup samples from manufacturer 1 **(A)** and 2 **(B)** were diluted and added to the cultures of *L. monocytogenes ΔpdeB/C/D* strain grown in the HTM/G medium for 48 h. Maple syrup grades are indicated by color.

Because NTG is partially soluble in water, we surmised that it may be present in maple xylem sap and in maple syrup, which is derived from the sap via boiling using open pan heating, sometimes combined with reverse osmosis (Saraiva et al., 2022). To test the antibiofilm activity of maple syrup, we purchased maple syrup from manufacturers in Vermont and New York, the two largest maple syrup producer states in the USA (USDA, 2023). The selection criteria were that syrup was declared to be free of additives, i.e., 100% pure, and that several varieties were available from the same manufacturer. All maple syrup varieties, Amber, Dark and Very dark from manufacturer one (Figure 6A), and Golden, Amber and Dark from manufacturer two (Figure 6B) possessed potent *L. monocytogenes* antibiofilm activity. The syrup samples with the highest activity inhibited clumping in the *ΔpdeB/C/D* strain after being diluted 1:800, and the samples with lowest activity – 1:200. While some variability in antibiofilm activity among samples was observed, small number of tested samples precludes us from concluding if certain syrup grades contain higher antibiofim activity than others. Interestingly, the extent of inhibition of EPS-biofilms depended on maple syrup concentration in a nonlinear manner suggesting that relatively high syrup concentrations (dilutions 1:50 to 1:100) may actually stimulate bacterial growth or biofilms. Overall, these results show that the *L. monocytogenes* antibiofilm activity is present at high levels in various kinds of maple syrup.

### Lariciresinol, an abundant lignan in maple syrup, possesses listerial antibiofilm activity

**Figure 7:**
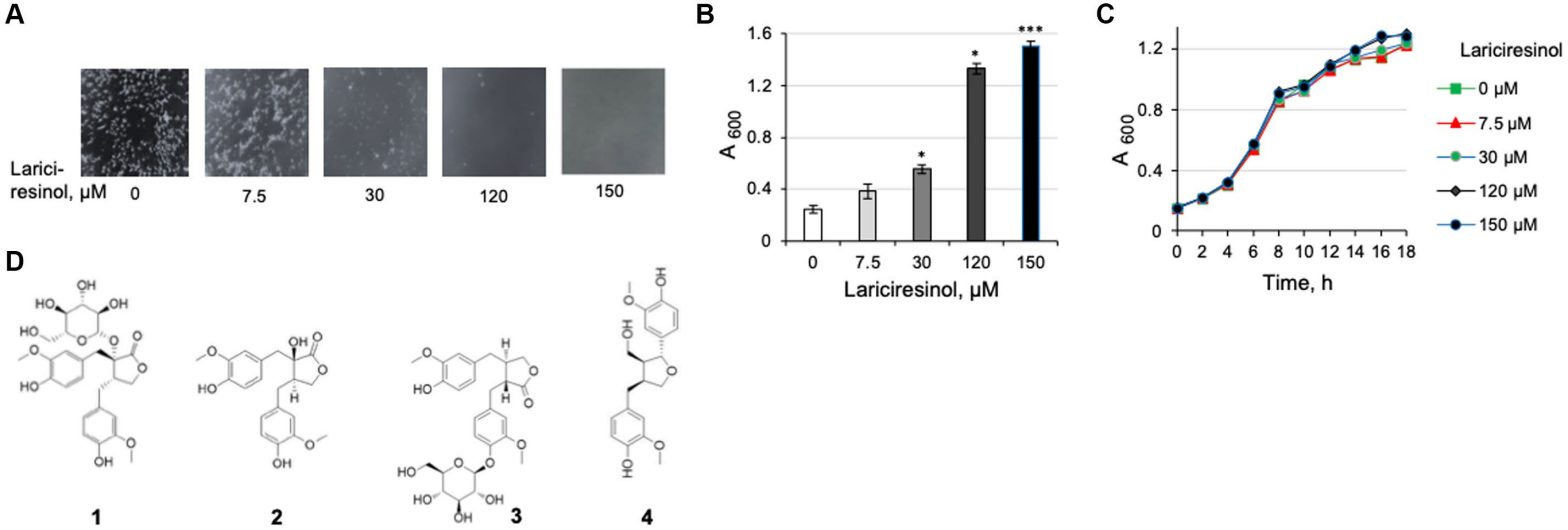
Characterization of lariciresinol as an antibiofilm compound from maple syrup. **(A)** Dose-dependent inhibition of *L. monocytogenes ΔpdeB/C/D* clumping by lariciresinol. **(B)** Quantification of the effect of lariciresinol via a clump precipitation assay. **(C)** Growth curves of *L. monocytogenes* EGD-e in the presence of lariciresinol showing that it does not impair listerial viability. Conditions used are the same as in Figure 4. **(D)** Structures of lignans (taken from www.chemfaces.com) tested for listerial antibiofilm activity. 1, NTG; 2, NT; 3, matairesinoside, 4, lariciresinol.

While NTG was identified as an abundant lignan in the methanol extracts from maple wood, it was not reported among chemicals identified in maple syrup (Ramadan et al., 2021). We wondered if an additional compound(s) in syrup possesses the *L. monocytogenes* antibiofilm activity. Lariciresinol, the most abundant lignan reported in maple syrup (Ramadan et al., 2021), was an obvious chemical to test. We found lariciresinol to possess antibiofilm activity at levels comparable to those of NTG (Figures 7A,B). Like NTG, lariciresinol did not impact *L. monocytogenes* viability (Figure 7C). We therefore conclude that at least two maple lignans have *L. monocytogenes* antibiofilm activity.

To investigate whether structurally related lignans possess antibiofilm activity, we tested nortrachelogenin (NT) and matairesinoside, which, to our knowledge, have not been detected in maple extracts. NT, also known as wikstromol and pinopalustrin, differs from NTG by the lack of glucoside moiety. Matairesinoside contains the NT core and is a glucoside, like NTG, yet its glucose moiety is linked in a different position than in NTG (Figure 7D). We found that, unlike NTG, NT inhibits *L. monocytogenes* growth, at 20 μM. The antibacterial activity of NT has been observed earlier and associated with its perturbing cytoplasmic membrane integrity (Lee et al., 2016). Matairesinoside, at 120 μM, was neither toxic nor possessive of the antibiofilm activity. The high specificity of action, compared to the structurally related lignans, suggests that NTG and lariciresinol interact with a specific target(s) in *L. monocytogenes* that affects EPS synthesis and/or dispersion. The identity of this target remains to be elucidated.

## Discussion

The emergence of contaminated fresh produce as a major source of listeriosis outbreaks in the past few decades is concerning. This concern is likely to grow because the consumption of raw fruit and vegetables is rising due to their increased affordability and healthier diet choices made by the public. Another contribution to the increasing severity of listeriosis outbreaks in the developed and some developing nations may be due to the growing share of people who are most susceptible to listeriosis, i.e., the elderly and the immune compromised individuals. To avoid fresh produce-based listeriosis outbreaks, we need affordable means to prevent listerial colonization of the surfaces of fruit and vegetables and to disable factors that help listeria persist post-colonization.

One factor whose role in fresh produce safety may have been underestimated is listerial EPS. While many bacterial species use EPS for plant surface attachment followed by the formation of EPS-containing biofilms (Danhorn and Fuqua, 2007; Yaron and Römling, 2014; Limoli et al., 2015; Castiblanco and Sundin, 2016), only recently has this been revealed in *L. monocytogenes* (Fulano et al., 2023). The Pss EPS synthesized by *L. monocytogenes* (i) increases colonization of the surfaces of various kinds of fresh produce, particularly rough surfaces like cantaloupe rind, (ii) improves the tolerance of the EPS-based biofilms to desiccation experienced by listeria during produce storage and transportation, and (iii) enhances acid stress resistance, which may improve listerial survival during exposure to the stomach acid following consumption of the contaminated produce. According to our estimates, when combined, these advantages increase the chance of EPS-synthesizing strains of reaching the small intestines of consumers by 10^2^ to 10^4^-fold, compared to the nonsynthesizing strains (Fulano et al., 2023). Importantly, disinfection alone may be insufficient for eradicating listeria (Fagerlund et al., 2020) because, compared to planktonic bacteria, listeria in EPS-biofilms are drastically, by 10^2^ to 10^6^-fold, more tolerant of disinfectants including quaternary ammonium salts and sodium hypochlorite (Chen et al., 2014). The addition of antibiofilm agents to cleaning and disinfection protocols may be necessary for eliminating listerial EPS-biofilms (Oloketuyi and Khan, 2017).

In this study, we serendipitously discovered potent *L. monocytogenes* antibiofilm activity of aqueous wood extracts of the *Acer* and *Carya* representatives and focused on identifying active compounds from maple (Figures 1–3). Two lignans, NTG and lariciresinol, previously detected in maple wood and maple syrup, respectively, inhibit EPS formation (Figures 4, 5, 7A,B). At 120 μM, NTG decreased the number of EPS-synthesizing bacteria on the surface of cantaloupe by >150-fold, and on cut celery and lettuce — by >10-fold (Figure 5A). Unexpectedly, maple lignans not only inhibited colonization of fresh produce by the EPS-synthesizing strain, but also decreased colonization by the strains that do not synthesize measurable levels of EPS under our experimental conditions, including four strains from the infamous 2011 listeriosis outbreak (McCollum et al., 2013). These results broaden the applicability of maple products to many, if not all, *L. monocytogenes* strains.

The antibiofilm activity of NTG and lariciresinol contrasts the lack of such activity in a chemically similar matairesinoside (Figure 7D), indicating that maple lignans act via a specific target in *L. monocytogenes*. Currently, we do not know if the antibiofilm activity of maple products is limited to the two identified lignans and if they act via the same target. Additional lignans have been reported in maple syrup and maple sugar, another food product of maple sap evaporation (Li and Seeram, 2010, 2011; Zhang et al., 2014; Liu et al., 2017). It is possible that some of these lignans or non-lignan compounds also have antibiofilm activity. Moreover, this possibility seems likely given that the reported lariciresinol concentration in maple syrup [approximately 54 μM (St-Pierre et al., 2014)], is much lower than the concentrations of pure lariciresinol needed for antibiofilm activity (Figures 7A,B). The presence of NTG in maple syrup was not reported, which suggests that it is not a major lignan there. In the follow-up studies we intend to address the mechanisms of action of maple compounds, identify their targets in *L. monocytogenes*, and explore the possibility of synergistic effects on biofilm inhibition. Below, we would like to address potential applications of our findings for enhancing fresh produce safety.

Maple trees grow in many parts of North America, Asia, Europe and northern Africa, and maple products are widely used in construction, furniture and food industries (Bi et al., 2016). Waste from maple lumber processing in the form of chips, shavings, sawdust and bark is reasonably available. Aqueous extracts of such waste can perhaps be added to water used for fresh produce washing. In our experiments, 1:10 dilutions of aqueous maple extracts made by soaking 1 g maple chips in 10 mL water, completely inhibited listerial biofilm formation on fresh produce of various kinds (Figure 2). Further, maple sap or syrup may serve as abundant sources of listerial antibiofilm agents. Maple syrup is a common food sweetener produced in millions of gallons per year (Statistics Canada, 2023; USDA, 2023). In our experimental setup, maple syrup inhibited listerial biofilms after being diluted 1:200 to 1:800 (Figure 6). Syrup is an approximately 20 to 50-fold concentrated sugar or red maple sap (Ramadan et al., 2021). Assuming that antibiofilm products are not destroyed during sap evaporation (Gerstenmeyer et al., 2013), we can estimate the low-end antibiofilm compound concentration in maple sap as (200/50=) 4 × and the high-end as (800/20=) 40 ×. A potential problem in using maple sap collected for syrup production is high levels of sucrose, 1–4%. Even after a 4 to 40-fold dilution, sucrose levels may remain sufficiently high to promote the growth of harmful fungi or bacteria on the sap-treated produce. One way to avoid high-sugar content is to collect sap from those maple species that naturally have much lower sucrose levels than the sap from sugar and red maple (Ramadan et al., 2021). Another way is to collect maple sap after the regular harvesting season because the levels of sugars in maple sap then drop drastically (Wong et al., 2005). Maple sap collection after the regular harvesting season would yield a low-sugar content sap for the antibiofilm use with no disruptions to syrup production.

It is worth noting that maple products exhibit the highest antibiofilm effect on the *L. monocytogenes* strain, *ΔpdeB/C/D*, that synthesizes copious amounts of the Pss EPS. At present, we do not know to what extent the Pss EPS poses a problem in fresh produce industries – partly because we poorly understand conditions promoting Pss EPS synthesis in *L. monocytogenes*, partly because we lack molecular tools for Pss detection, and partly because the role of EPS in fresh produce colonization had only recently been uncovered (Fulano et al., 2023). However, from the standpoint of fresh produce safety, the 10^2^ to 10^4^-fold advantage that EPS may render *L. monocytogenes* in causing foodborne illness is difficult to ignore, even if EPS-biofilms are formed only by a subset of *L. monocytogenes* strains, under a subset of conditions. Therefore, developing maple products into antibiofilm agents that can be added to the existing produce washing, cleaning and disinfection practices may be worthwhile. Importantly, as uncovered in this work (Figures 5B,C), such products will likely inhibit colonization by all *L. monocytogenes* strains even if they do not synthesize EPS.

## Materials and methods

### Bacterial strains and growth conditions

This study used *L. monocytogenes* EGD-e (wild type, ATCC BAA-679*) and its high-c-di-GMP derivative, *ΔpdeB/C/D*, that contains in-frame deletions in three c-di-GMP phosphodiesterase genes, *pdeB*, *pdeC* and *pdeD* (Chen et al., 2014). Four strains from the 2011 cantaloupe-associated listeriosis outbreak, L2624, L2625, L2626 and L2476, were a gift from Dr. Knabel, Pennsylvania State University. All strains were grown in the liquid minimum HTM medium (Tsai and Hodgson, 2003) containing 3% glucose, HTM/G, at 30°C under shaking. Biofilm formation on wood coupons and fresh produce pieces was assessed after incubation for 48 h. The effects of maple compounds on the growth, A_600_, of the wild-type strain, EGD-e, were monitored by inoculating 1:100 dilution of the overnight *L. monocytogenes* culture in the HTM/G medium containing maple compounds. For enumerating CFUs, cultures were plated onto Brain Heart Infusion (BHI) agar (Millipore Sigma) and incubated at 37°C for 36 h.

### Wood products and phytochemicals

The wood products were purchased on Amazon.com from various vendors. The following wood coupons/disks (sold for do-it-yourself arts and crafts projects) were used here: poplar disks, 50 mm diameter × 2.5 mm thickness,^1^ and maple disks, 19 mm diameter x 3.2 mm thickness.^2^ Wood chips for preparing aqueous extracts were from Camerons (Smoker Wood Chips, Variety Gift Set), CO, USA. Maple syrup from these sources was used: (1) Classic Trio Collection featuring Amber, Dark, and Very Dark color by Crown Maple (NY, USA) and (2) Maple Syrup Grading Sampler (Grade A) containing Golden, Amber and Dark color samples by Butternut Mountain Farm, Maple Sugar Company (VT, USA). A *T. jasminoides* (star jasmine) plant was purchased from Fast Growing Trees Nursery (SC, USA). Phytochemicals were purchased from the following suppliers: fraxetin and procyanidin A2 from Alkemist Labs (CA, USA), epicatechin gallate, quercetin 3-gentiobioside, and quercetin 3-O-β-D-xylopyranoside from Aobius Inc. (MA, USA), quercetin 3-D-galactoside and phlorizin from Cayman Chemical Company (MI, USA), matairesinoside from ChemSpace (NJ, USA), catechin and epicatechin from Sigma-Aldrich (MO, USA), and nortrachelogenin, nortrachelogenin-8’-O-b-D-glucopyranoside, and lariciresinol from Targetmol (MA, USA). Sugars (sucrose, raffinose, stachyose, glucose, fructose, xylose), organic acid salts (malate, citrate, succinate), and simple phenolics were purchased from Sigma-Aldrich (MO, USA).

### Preparation and treatment of wood coupons and pieces of fresh produce used in biofilm experiments

Overnight *L. monocytogenes* cultures were diluted 1:100 into 10 mL HTM/G medium in 125-mL flasks and grown at 30°C until optical density, A_600_, ~ 0.4, at which point sterile wood coupons or pieces of fresh produce were added, and cultivation was continued for 48 h. Whole cantaloupes, celery, Iceberg lettuce, strawberries, peaches, bell peppers, carrots and sweet potato were purchased from local (Laramie, WY) retail stores. The wood coupons were autoclaved (121°C, 30 min). Fresh produce was thoroughly washed, dried and cut into approximately equal pieces. Cantaloupe coupons (20 mm diameter × 4 mm thickness) were obtained by using a cork borer. Celery and Iceberg lettuce from similar sized stocks/leaves were cut in pieces of ~20 mm in length. These pieces were sterilized by extended exposure to sodium hypochlorite followed by extensive washing from the traces of the hypochlorite, as described earlier (Fulano et al., 2023). Following incubation for 48 h, produce pieces were aseptically withdrawn, rinsed in HTM/G twice to remove loosely bound biofilms, and mechanically macerated in the homogenizer (Stomacher^®^ 80 Biomaster, Seward, UK), as described earlier (Fulano et al., 2023). The serial dilutions of the homogenates were plated onto BHI agar plates for CFU enumeration.

Aqueous wood extracts were prepared by soaking autoclaved wood coupons, chips or dried and mechanically disintegrated stems for 24 h, using 1 g wood per 10 mL sterile water. Extracts were added at 1 mL per 10 mL listerial culture, unless indicated otherwise.

### *Listeria monocytogenes* clump precipitation assay

Clump precipitation assay was used to quantify the antibiofilm activity of maple compounds. Overnight cultures of *L. monocytogenes ΔpdeB/C/D* were diluted into fresh HTM/G medium amended with test compounds to initial A_600_, 0.16. Ten mL cultures in 125-mL flasks were shaken (120 rpm) for 48 h at 30°C. Samples of cultures were then transferred to the 1-mL spectrophotometer cuvettes to record A_600_ exactly after 2 min from the time of transfer. This time was sufficient for the intact, nondispersed, EPS-clumps to precipitate to the bottom of the cuvette, where they do not contribute to the optical density, A_600_, measurements. The A_600_ of the suspended bacterial culture is reported as a measure of clump dispersion. The higher the antibiofilm activity, the less amount of clumps are present in the culture, i.e., the higher its A_600_ measured after clump precipitation.

### Statistical analysis

Microsoft Excel was used for data processing and analysis. The bar charts display a mean ± standard deviation (SD) from three independent experiments, each performed in at least two replicates. Unpaired Student’s *t*-tests were performed using Prism 9 for Mac (GraphPad). Significant differences, compared to the no-treatment controls, are indicated in all figures as follows: *p < 0.05; **p < 0.01; ***p < 0.001, and ****p < 0.0001.

## Data availability statement

The raw data supporting the conclusions of this article will be made available by the authors, without undue reservation.

## Author contributions

AE: Data curation, Formal analysis, Investigation, Methodology, Visualization, Writing – original draft. AF: Data curation, Formal analysis, Investigation, Methodology, Visualization, Writing – original draft. MG: Conceptualization, Funding acquisition, Methodology, Project administration, Resources, Supervision, Writing – original draft, Writing – review & editing.

## Funding

The author(s) declare financial support was received for the research, authorship, and/or publication of this article. This work was supported by USDA-NIFA-AFRI-2020-67014-32496 and in part by the NIFA HATCH Program via the University of Wyoming Agriculture Experimental Station grant WYO-00635-20.

## Footnotes

^1^ www.Juvale.com

^2^ www.Toyingaround.etsy.com
